# Supplementary material for: In Vitro Evaluation and Mitigation of Niclosamide’s Liabilities as a COVID-19 Treatment
Source: Vaccines (Basel). 2022 Aug 9;10(8):1284. doi: 10.3390/vaccines10081284 (PMC9412300; doi:10.3390/vaccines10081284)
Supplement: Supplementary file 1 [file vaccines-10-01284-s001.zip › vaccines-1827606-SI.pdf]

Supplemental Figures

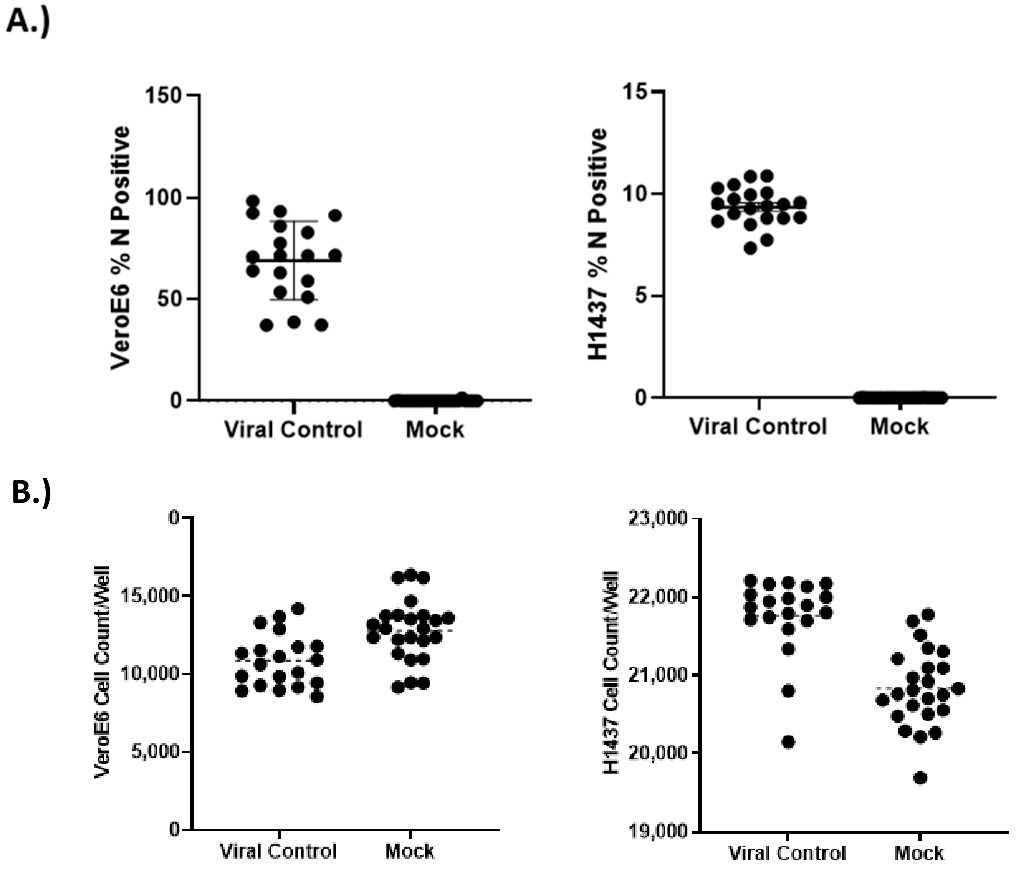

Supplemental Figure S1. Control data for VeroE6 and H1437 SARS-CoV-2 long-term exposure bioassays. Data were analyzed in GraphPad Prism 9.0 and is shown as mean ± standard deviation for *n* = 20 replicates per condition. A.) Raw percent N protein positivity. Average percent infection was 69% for VeroE6 and 9% for H1437. This data was used to determine a normalized percent infection for treated wells. B.) Cell observations per well. Viral control data was used to determine a percent viability score for treated wells.

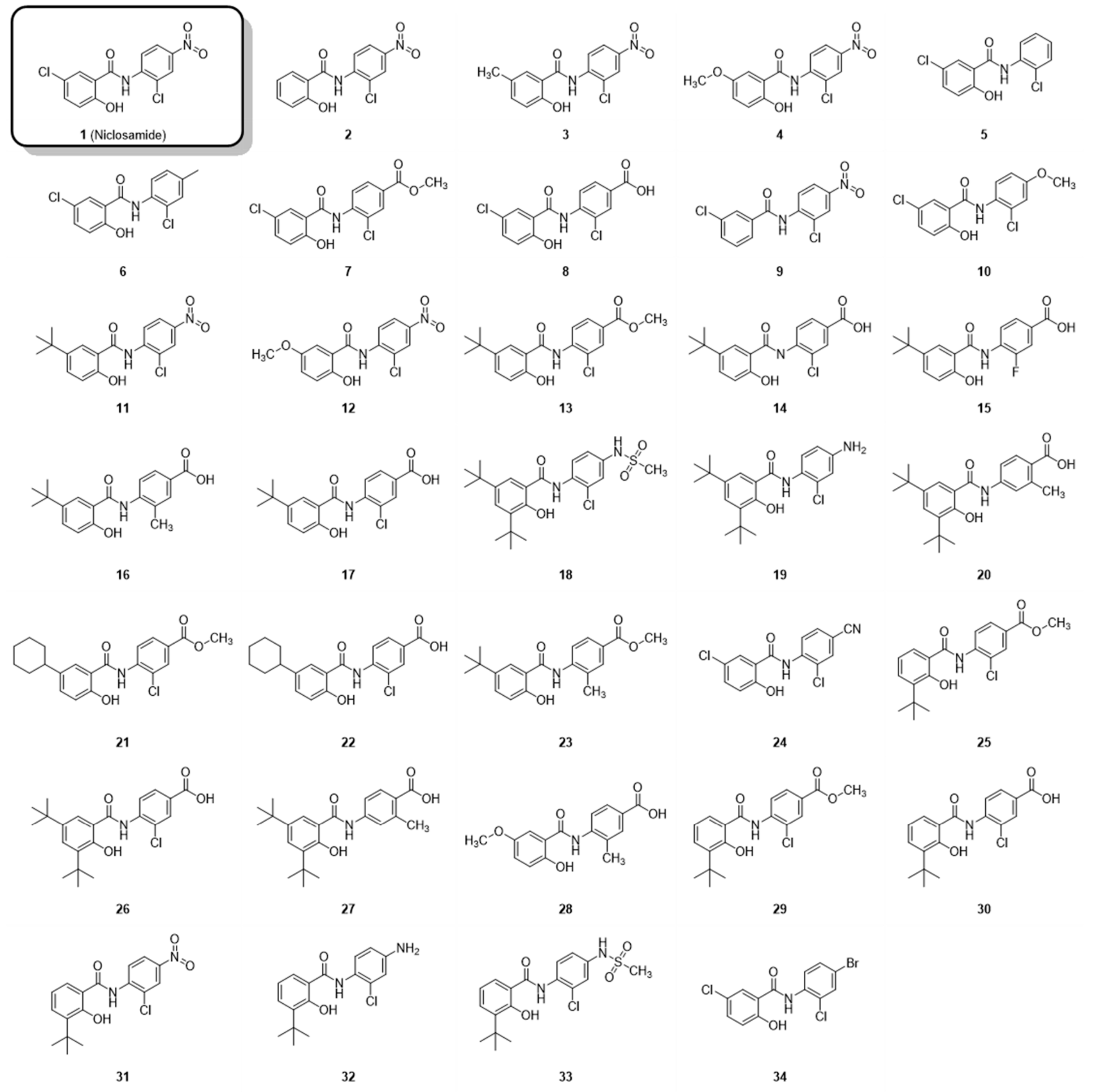

Supplementary Figure S2. Niclosamide analog structures. Modifications were made to the chlorosalicylic acid and/or nitroaniline rings. Compound number is indicated below the structure. Structures were generated using ChemDraw version 21.0 (PerkinElmer, Waltham, Massachusetts, United States).

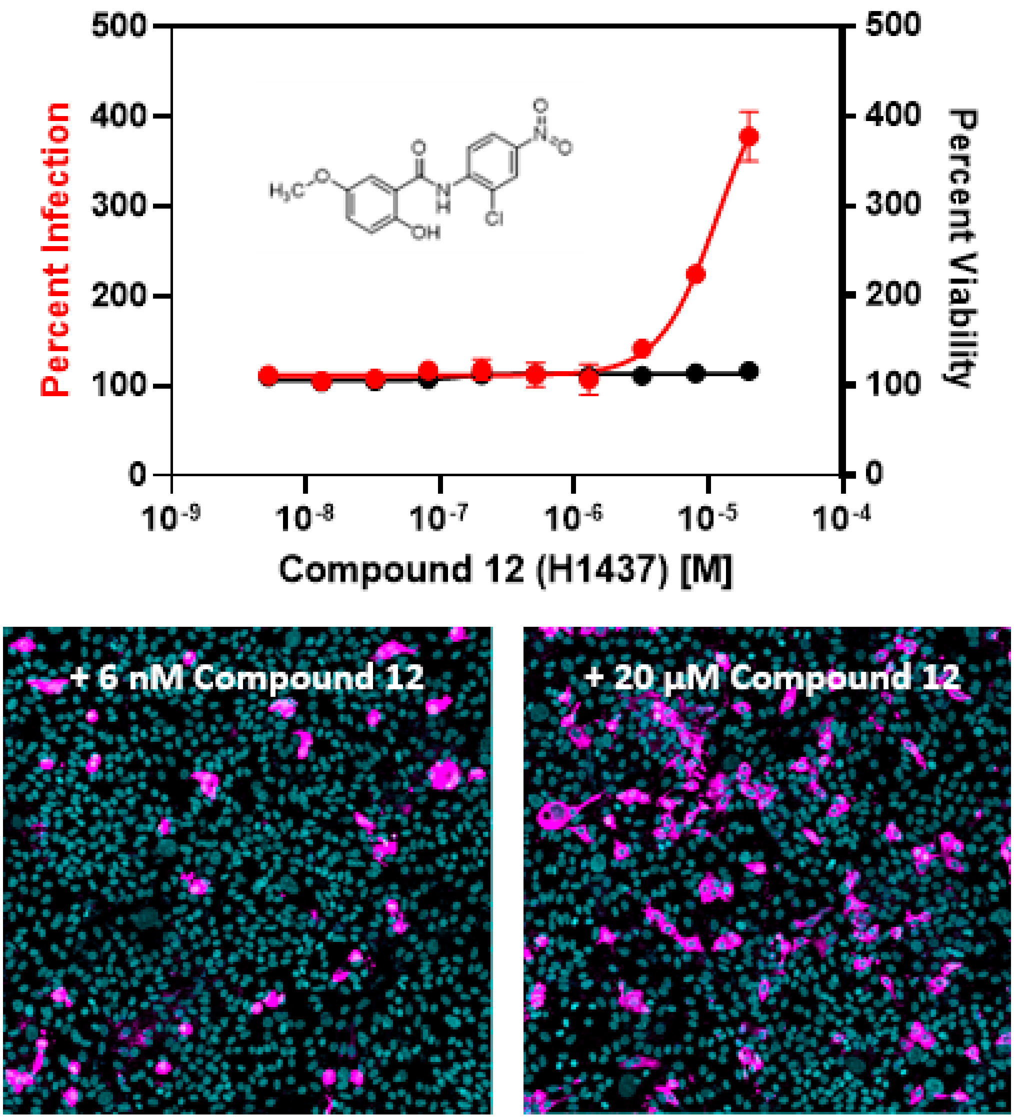

Supplemental Figure S3. Some niclosamide analogs cause dose-dependent exacerbation of infection in H1437. As a representative example, compound 12 causes a 400% increase in infection at 20 µM. A concentration-response curve for infection is shown here along with representative images for a non-exacerbating concentration (6 nM) and an exacerbating concentration (20 µM). Images were captured at 20X magnification using a Yokogawa CV8000

high content imaging microscope and processed using ImageJ. Nuclei are colored in cyan and SARS-CoV-2 N protein is colored in magenta.
